# Supplementary material for: Genome-wide sequencing identifies a thermal-tolerance related synonymous mutation in the mussel, Mytilisepta virgata
Source: Commun Biol. 2023 Jan 3;6:5. doi: 10.1038/s42003-022-04407-4 (PMC9810668; doi:10.1038/s42003-022-04407-4)
Supplement: Supplementary file 2 — Electronic supplementary material [file 42003_2022_4407_MOESM2_ESM.pdf]

Genome-wide sequencing identifies a thermal-tolerance related synonymous mutation in the mussel, *Mytilisepta virgata*

Short title: Synonymous mutations associated pwith heat tolerance、

Authors: Yue Tan[1†], Chaoyi Ma[1†], Xiaoxu Li[1], Guodong Han[2], Yunwei Dong[1, 3 *]

1. The Key Laboratory of Mariculture, Ministry of Education, Fisheries College, Ocean University of China, Qingdao 266003, PR China
2. College of Life Science, Yantai University, Yantai, 264005, China
3. Function Laboratory for Marine Fisheries Science and Food Production Processes, Pilot National Laboratory for Marine Science and Technology, Qingdao 266235, PR China

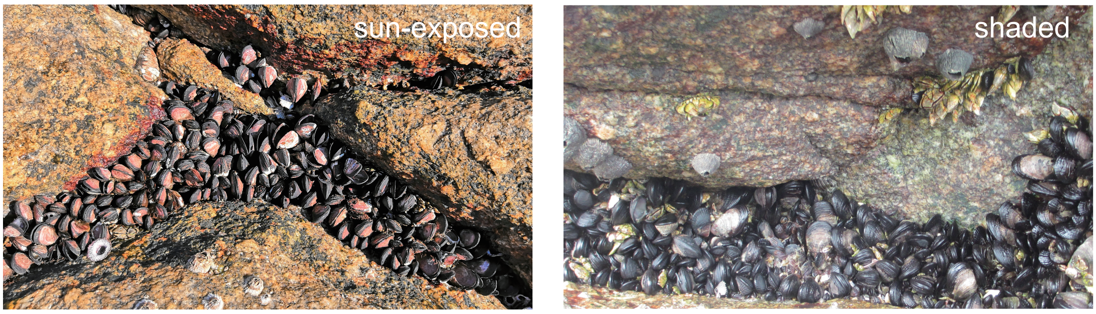

Supplementary Fig. 1 | Photos for *M. virgata* individuals in sun-exposed and shaded microhabitats.

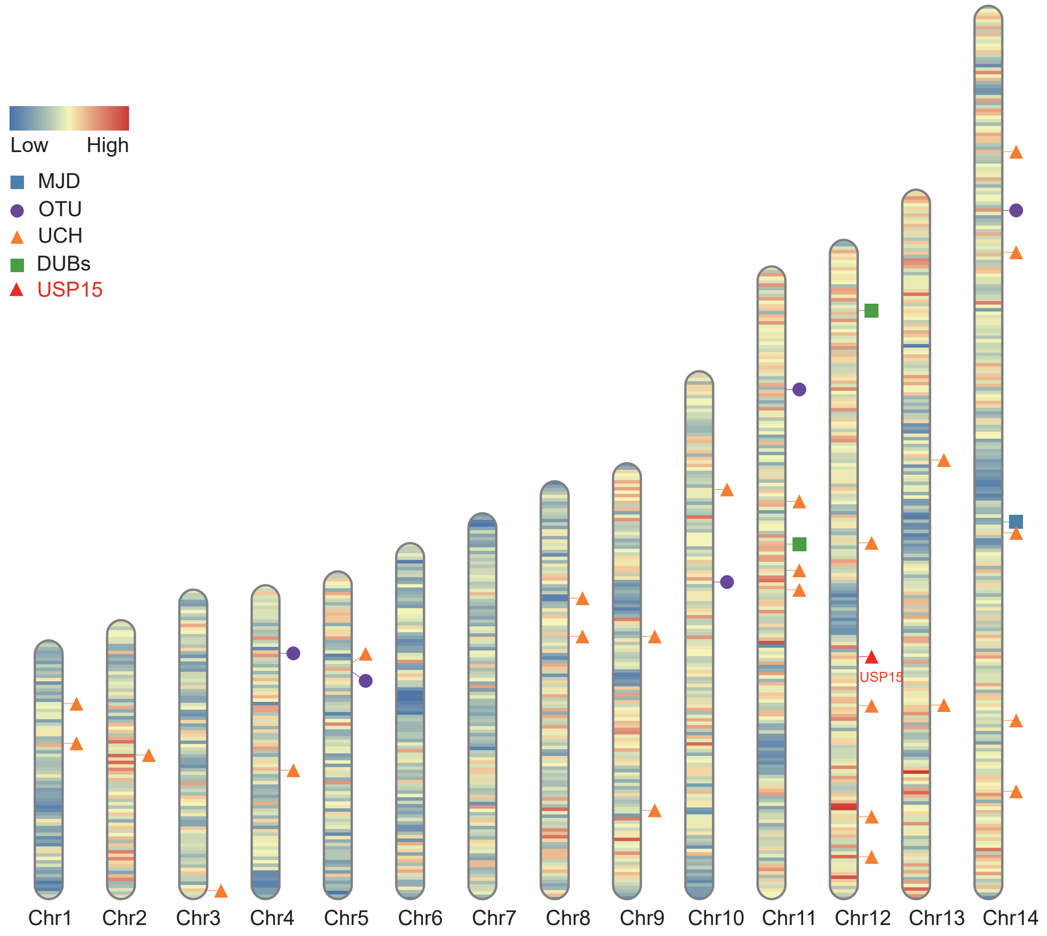

Supplementary Fig. 2 | Genome-wide distribution of subfamilies of Deubiquitinating enzymes (DUBs). The overlaid heatmap shows the gene density (shown in gradient color) and the tack labels refer to genes belonging to certain subfamilies of Deubiquitinating enzymes (DUBs) found across the *Mytilisepta virgata* genome [1], which consist of Josephin domain-containing (MJD) proteases shown in the blue box, Otubain domain ubiquitin binding proteins (OTU) shown in purple circles, ubiquitin C-terminal hydrolases (UCH) shown in orange triangles and two genes belonging to DUBs but without exact annotation to any subfamilies shown as DUBs in the green box. *MvUSP15* gene was shown in the red triangle at chromosome twelve.

atg gct gaa ggt ggt gtc tcg gaa tgc aat gct caa aat gcc att gat att gac acg gcg
M A E G G V S E C N A Q N A I D I D T A
gcc ctt gaa aag cag aaa aat gaa ttg aaa gat ttg ctg aag aag cca tta aag cgg gac
A L E K Q K N E L K D L L K K P L K R D
gac aaa tgg tat ctg ctt gat gca aaa tgg ttt aaa caa tgg aag aaa tat gtt ggc tat
D K W Y L L D A K W F K Q W K K Y V G Y
gaa agt tgg gac tcc ttc aat gtt gga cag tct gaa gca aat ccc ggt ccc ata gat aat
E S W D S F N V G Q S E A N P G P I D N
tct caa ttg ctt gat gag aat gga aaa ttg aag gag cac ctt ata gat gag tta gat tat
S Q L L D E N G K L K E H L I D E L D Y
caa ctg ttt cca gag gag gct tgg aat aag atc atg tcc tgg tat ggg ttg atg gaa ggt
Q L F P E E A W N K I M S W Y G L M E G
cag aca cca ata gag aga aaa gtg att gag caa ggg atg ttt gtc aaa cat tgc aaa gta
Q T P I E R K V I E Q G M F V K H C K V
gaa gtt tat ctt ctg gaa ata aaa atg tgt gaa aat tct aat cta gag aag atc att agc
E V Y L L E I K M C E N S N L E K I I S
aaa cag atg agt agg gct gaa act att gag gat ttg gag aag gaa ctg aga aaa aca ttt
K Q M S R A E T I E D L E K E L R K T F
gag ata gcg gaa gaa aag gaa gtg cga ctt tgg tat aaa tat atg agt aac aca tac gaa
E I A E E K E V R L W Y K Y M S N T Y E
cat ctt agt aaa aaa gat agt aca tta caa gat gct gca ttg tat tct gga cag gta att
H L S K K D S T L Q D A A L Y S G Q V I
gta ata gaa cag aaa aat gat gat ggg aca tgg cca aga caa gct aaa agt aca aac agt
V I E Q K N D D G T W P R Q A K S T N S
tac aac agt agt tca tac act tct aca agg tcc agt tca agt gga tat gat tcc ggg tat
Y N S S S Y T S T R S S S S G Y D S G Y
ggg tcg tcc tat ggt tac tat gga tac gac cag gga agg ggt ggc agt gtg gca cct gga
G S S Y G Y Y G Y D Q G R G G S V A P G
cta tgt ggc ctg tcg aat tta gga aac aca tgt ttt atg aat tca gca ata cag tgt atg
L C G L S N L G N T C F M N S A I Q C M
agt aat gtg cca aag tta aca gaa tat tta ctg agt gaa agg tgg att aat gaa gtg aat
S N V P K L T E Y L L S E R W I N E V N
gag agc aat cca ctt gga atg ggt ggt gaa ata gca agg tca tat gct gag ctg atc aag
E S N P L G M G G E I A R S Y A E L I K
gtc atg tgg tct gga aaa tac agt cat act gtt cct cga aat ttt aag gtg gct gta ggt
V M W S G K Y S H T V P R N F K V A V G
agg ttt gct cca cag ttt tct ggg tac cag caa cag gac tcc cag gaa tta ctg gct ttc
R F A P Q F S G Y Q Q Q D S Q E L L A F
tta tta gat ggc tta cat gaa gat ctg aac aga att cat aaa aaa cct tac ata gaa cag
L L D G L H E D L N R I H K K P Y I E Q
aaa gat gct gat gat cga tta gat gaa ctt gta gcc aag gaa tcc tgg gac aac tat aga
K D A D D R L D E L V A K E S W D N Y R
aaa agg aat gat tct gtg ata gtt gac act ttc cat gga cta ctc aaa tcc acg gta cat
K R N D S V I V D T F H G L L K S T V H

tgt ccg gaa tgt gat aaa ata tca gtg act ttt gat cct ttc tgt tat ctt tcc tta cct

C P E C D K I S V T F D P F C Y L S L P

ctc cct gtg aag aag gaa cga cag ttg gaa ata ttc tgg gtt cca ctt agt cct gag aaa

L P V K K E R Q L E I F W V P L S P E K

aaa cct gta cag ttc aaa tta ata gtg cca aag atg gga agt gtc tca gat atg tgt aaa

K P V Q F K L I V P K M G S V S D M C K

ata ttg tca gaa aaa gta gga gta gaa ccc ata aag atg gta gtt aca gat gtt tac aac

I L S E K V G V E P I K M V V T D V Y N

cat agg ttc cat aaa gtg ttc agt cca gaa gaa agc ctt agt tat att tta gat agg gat

H R F H K V F S P E E S L S Y I L D R D

gat att ttt gta tat gag gta cct gtg tcc aaa tca gat gat cct gaa act gtt gtt gta

D I F V Y E V P V S K S D D P E T V V V

cca att tat atg aga gaa aag aaa cca aga aac agt tac cat agt ggt tca tat caa ctt

P I Y M R E K K P R N S Y H S G S Y Q L

ttt ggt cag cct ctg ctg ctg cca gtg tcc aga aaa aat tgt aca tat gaa tct ttg tat

F G Q P L L L P V S R K N C T Y E S L Y

aac aca tta ctc cac aga ata tca cgt tat gtg aaa atg cct tca gaa gat gac aaa tgg

N T L L H R I S R Y V K M P S E D D K W

tgg atg gta gag aaa gat gga aaa atg gtg aat gat aac agt gat ggt gag aaa gat tca

W M V E K D G K M V N D N S D G E K D S

gaa gat gaa gat aaa aat gat gaa gaa atg gac act tcc agt gat acg aag gaa att cat

E D E D K N D E E M D T S S D T K E I H

cag aat gga gat ata aat gga gaa ctt aat ggt cat gac aaa aaa gct aaa cct tca cct

Q N G D I N G E L N G H D K K A K P S P

tta atg ttc aaa ttt act gtg gtg aat tca tat ggc agt aca gaa ata aac tac aaa ctg

L M F K F T V V N S Y G S T E I N Y K L

gat cga gat ggg gaa cca ctc aga tta aat agt agg aca tat gta gct gtg gac tgg aat

D R D G E P L R L N S R T Y V A V D W N

cca tta gct cag gaa aaa ttt tat gat gac aag gct gct gaa gat ttt gaa caa gat gag

P L A Q E K F Y D D K A A E D F E Q D E

agt atg aac tac aga gcc cag aag aaa caa att ata cag ctt ggt gat tgt ctg gat ttg

S M N Y R A Q K K Q I I Q L G D C L D L

ttt aca aaa gaa gaa cag cta gga gaa aat gac ttg tgg tat tgt cca aga tgt aaa aaa

F T K E E Q L G E N D L W Y C P R C K K

cat caa caa gct aca aaa aag ttt gac tta tgg tca ctg cca gac gtt ttg att att cac

H Q Q A T K K F D L W S L P D V L I I H

ctt aag aga ttc tcc tac aac aga tac ttc aga gac aaa ata gat gtg ata gta gaa ttc

L K R F S Y N R Y F R D K I D V I V E F

cct cca aga gga ttg agt atg aac aaa tac gtt atc aac gcc aac cat gga cct gct aat

P P R G L S M N K Y V I N A N H G P A N

tat gac ttg ata gcc gtc tcc aac cat tat gga ggt ctc ggt gga gga cat tac acg gct

Y D L I A V S N H Y G G L G G G H Y T A

tat ggc aag aac aaa gag aat agt gaa tgg tac tac ttt gat gat tct agt gta agt cct

Y G K N K E N S E W Y Y F D D S S V S P

tcg	gca	gag	gat	gat	gtc	gtg	tca	aag	gca	gcc	tat	gta	ctt	gtt	tat	caa	aaa	cgg	act
S	A	E	D	D	V	V	S	K	A	A	Y	V	L	V	Y	Q	K	R	T
cag	tgc	caa	gca	aat	tct	aag	tca	gcc	agt	agg	acg	atg	aat	gga	cag	tgt	gac	gag	gac
Q	C	Q	A	N	S	K	S	A	S	R	T	M	N	G	Q	C	D	E	D
atg	gaa	aca	taa																
M	E	T	*																

Supplementary Fig. 3 | Nucleotide and deduced amino acid sequences of the *MvUSP15* cDNA. Blue and yellow backgrounds distinguish the 21 exons.

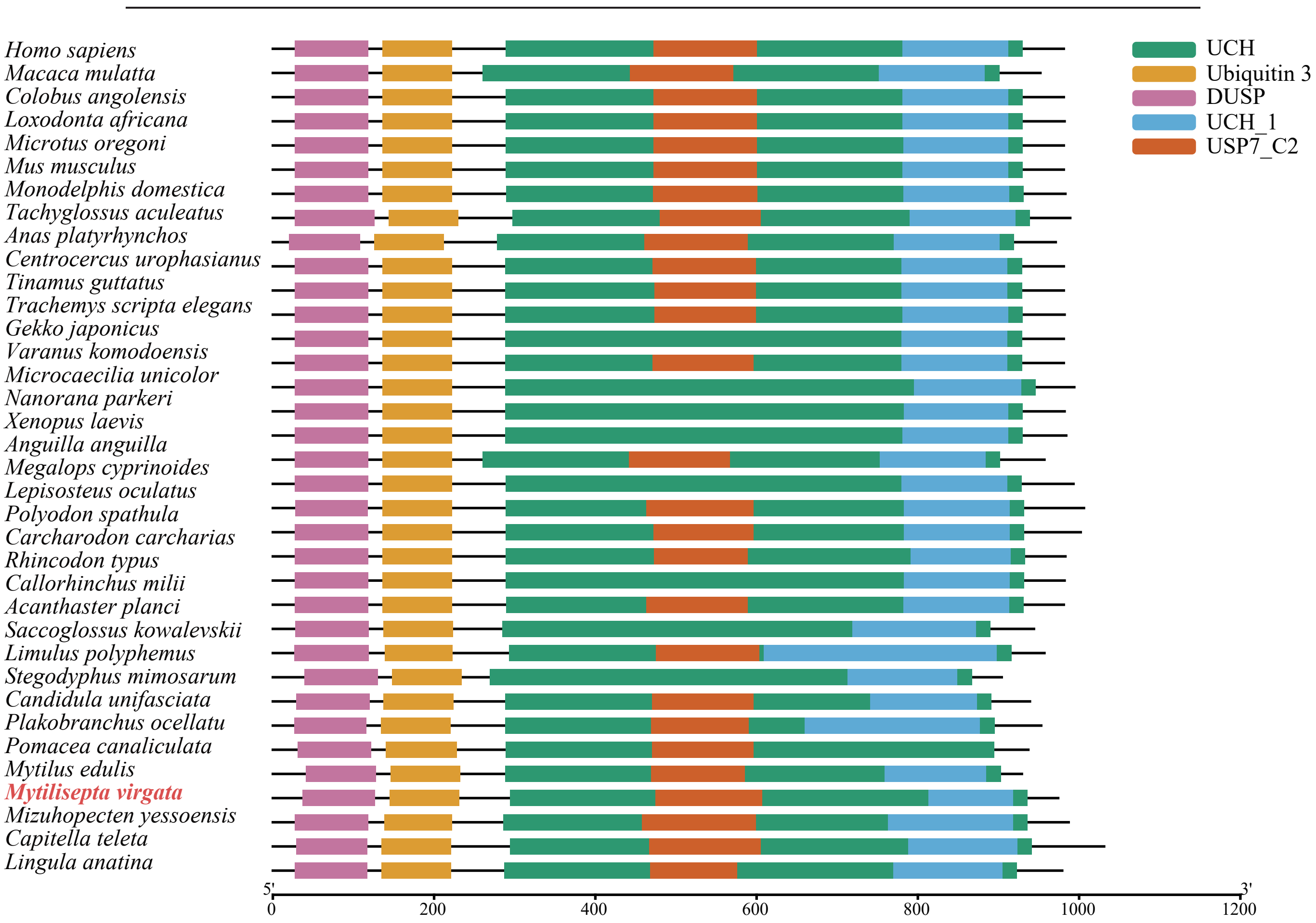

Supplementary Fig. 4 | Alignments of conserved domains in *USP15* protein from species belonging to different taxa.

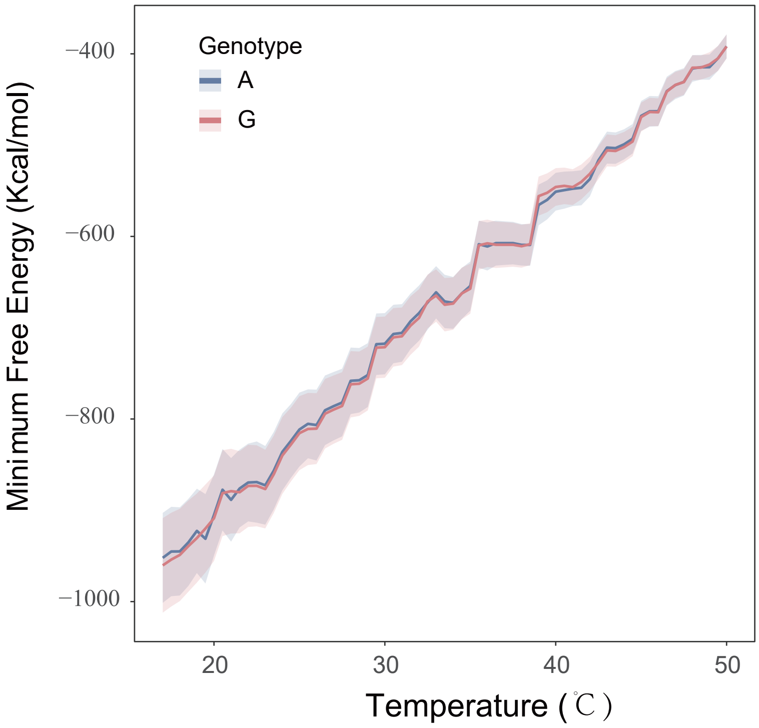

Supplementary Fig. 5 | Structural stability of two orthologous MvUSP15 mRNA sequence. Free energy change occurring during formation of the ensemble of secondary structures (ΔGfold) in two orthologous mRNAs with temperature rising. The red line represents the changes in ΔGfold of mRNA with G at the synonymous mutant site, while the blue line represents the changes in ΔGfold of mRNA with A. The red and blue shadow region represent the error estimates calculated by efn2 program for two orthologous mRNAs with G and A at the synonymous mutant site, respectively.

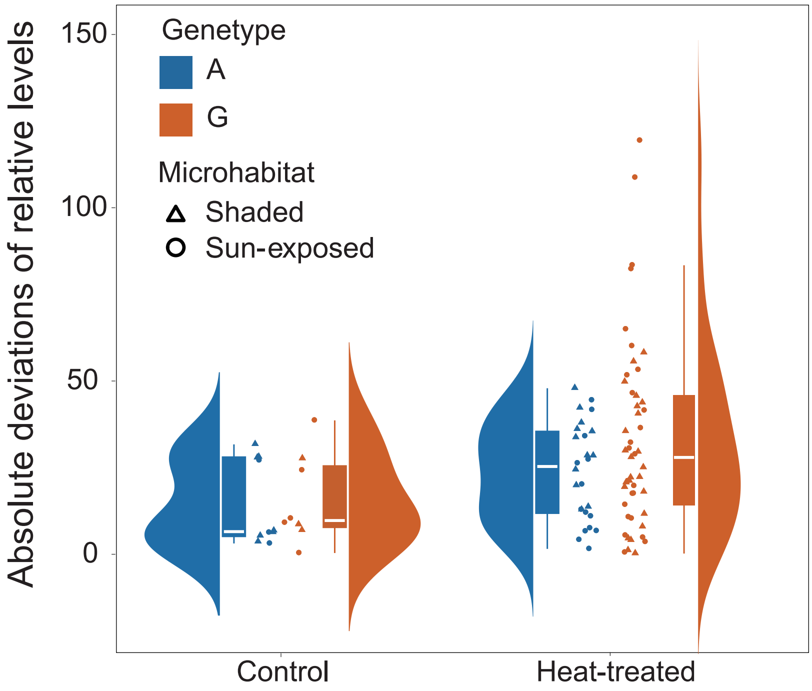

Supplementary Fig 6 | The profile of absolute deviations of *MvUSP15* expression levels in the mussels from the heat-treated group and control group. Different colors represent different genotypes. Each triangle and circle represent the absolute deviation of *MvUSP15* expression levels of an individual sampled from the shaded microhabitats and sun-exposed microhabitats, respectively.

Supplementary Table 1 | Two-factor ANOVA to measure the effect of genotype (G- or A-type) and the sampling month on ABT of *M. virgata*

Source of variation	SS	DF	F	*p*
Month	31.73	2	32.95	< 0.001
Genotype	37.70	1	78.28	< 0.001
Month × Genotype	1.16	2	1.20	0.311
Residuals	20.23	42		

Supplementary Table 2. The results of multiple comparisons using TukeyHSD analysis on ABT of four clusters.

Two clusters for comparison	Difference in means	*p* value after adjustment
2-1	-2.71	< 0.001**
3-1	-0.46	0.47
4-1	-2.51	< 0.001**
3-2	2.44	< 0.001**
4-2	0.19	0.92
4-3	-2.05	< 0.001**

Supplementary Table 3 | Protein domain architectures and features of *MvUSP15*

Name	Start residue	End residue	E-value
DUSP	36	132	$9.25e^{-34}$
Pfam: Ubiquitin_3	146	233	$7.2e^{-28}$
Pfam: UCH	282	896	$4.1e^{-78}$

Supplementary Table 4 | The results of multiple comparisons using TukeyHSD analysis on *MvUSP15* expression levels of five clusters.

Two clusters for comparison	Difference in means	*p* value after adjustment
2-1	-5.05	0.98
3-1	-16.29	0.50
4-1	83.53	< 0.001**
5-1	-7.44	0.96
3-2	-11.24	0.68
4-2	88.58	< 0.001**
5-2	-2.40	0.99
4-3	99.82	< 0.001**
5-3	8.84	0.89
5-4	-90.98	< 0.001**

Supplementary Table 5 | Primer pairs were used in this research. The pair EF-1α was from a previous study [2].

Primer pair name	Forward primer (5′ - > 3′)	Reverse primer (5′ - > 3′)
uch-CDS	ATGGCTGAAGGTGGTGTCTC	TTATGTTTCCATGTCCTCGTCA
GT-454	TAAGGTCTGTTGTCTCATCCA	TATTACTAAATCTTCCTCACTCAT
Qpcr1	CCAGACGTTTTGATTATTCA	ACTATTCTCTTTGTTCTTGCC
EF-1α	CTCTTCGTCTCCCACTCCAG	ACCAGGGAGAGCTTCAGTCA

References

1. Georges A, Gros P, Fodil N. 2021 USP15: a review of its implication in immune and inflammatory processes and tumor progression. *Genes Immun.* **22**, 12-23. (doi: 10.1038/s41435-021-00125-9)

2. Gerdol M, Fujii Y, Hasan I, Koike T, Shimojo S, Spazzali F, Yamamoto K, Ozeki Y, Pallavicini A, Fujita H. 2017 The purplish bifurcate mussel *Mytilisepta virgata* gene expression atlas reveals a remarkable tissue functional specialization. *BMC Genom.* **18**. (doi: 10.1186/s12864-017-4012-z)
