# Supplementary material for: Genome-wide sequencing identifies a thermal-tolerance related synonymous mutation in the mussel, Mytilisepta virgata
Source: Commun Biol. 2023 Jan 3;6:5. doi: 10.1038/s42003-022-04407-4 (PMC9810668; doi:10.1038/s42003-022-04407-4)
Supplement: Supplementary file 3 — Description of Additional Supplementary Files [file 42003_2022_4407_MOESM3_ESM.pdf]

Description of Additional Supplementary Files

File name: Supplementary Data 1

Description: In situ operative temperature of sun-exposed and shaded microhabitats in July and August 2020.

File name: Supplementary Data 2

Description: The GenBank sequence version number, scientific name of species, and

sequence of the USP15 proteins from different taxa.

File name: Supplementary Data 3

Description: The genotypes of 179 mussels in the heat-treated group and control group.

File name: Supplementary Data 4

Description: The raw expression data of MvUSP15 of the mussels used for determining the mRNA levels.

File name: Supplementary Data 5

Description: The source data underlying (a) Figure 1a, (b) Figure 2a, (c) Figure 2b, (d) Figure 2c, (e) Figure 4, (f-g) Supplementary Fig.2, (h) Supplementary Fig.5, (i) Supplementary Fig.6
