# Supplementary material for: Genome-wide sequencing identifies a thermal-tolerance related synonymous mutation in the mussel, Mytilisepta virgata
Source: Commun Biol. 2023 Jan 3;6:5. doi: 10.1038/s42003-022-04407-4 (PMC9810668; doi:10.1038/s42003-022-04407-4)
Supplement: Supplementary file 6 — nr-reporting-summary_COMMSBIO-22-1450B [file 42003_2022_4407_MOESM6_ESM.pdf]

nature portfolio

Corresponding author(s): Yunwei Dong, COMMSBIO-22-1450B

Last updated by author(s): Dec 10, 2022

Reporting Summary

Nature Portfolio wishes to improve the reproducibility of the work that we publish. This form provides structure for consistency and transparency in reporting. For further information on Nature Portfolio policies, see our Editorial Policies and the Editorial Policy Checklist.

Statistics

For all statistical analyses, confirm that the following items are present in the figure legend, table legend, main text, or Methods section.

n/a	Confirmed	
☐	☒	The exact sample size (*n*) for each experimental group/condition, given as a discrete number and unit of measurement
☐	☒	A statement on whether measurements were taken from distinct samples or whether the same sample was measured repeatedly
☐	☒	The statistical test(s) used AND whether they are one- or two-sided<br>*Only common tests should be described solely by name; describe more complex techniques in the Methods section.*
☒	☐	A description of all covariates tested
☐	☒	A description of any assumptions or corrections, such as tests of normality and adjustment for multiple comparisons
☐	☒	A full description of the statistical parameters including central tendency (e.g. means) or other basic estimates (e.g. regression coefficient) AND variation (e.g. standard deviation) or associated estimates of uncertainty (e.g. confidence intervals)
☐	☒	For null hypothesis testing, the test statistic (e.g. *F*, *t*, *r*) with confidence intervals, effect sizes, degrees of freedom and *P* value noted<br>*Give P values as exact values whenever suitable.*
☒	☐	For Bayesian analysis, information on the choice of priors and Markov chain Monte Carlo settings
☒	☐	For hierarchical and complex designs, identification of the appropriate level for tests and full reporting of outcomes
☒	☐	Estimates of effect sizes (e.g. Cohen's *d*, Pearson's *r*), indicating how they were calculated

Our web collection on statistics for biologists contains articles on many of the points above.

Software and code

Policy information about availability of computer code

Data collection	ddRADseq data (BioProject ID: PRJNA517974; BioSample accessions: SAMN10849586 - SAMN10849649);<br>Mytilisepta virgata genome assembly (BioProject ID: PRJNA910323)
Data analysis	The bioinformatics analysis were described in the methods. All calculation and comparative analysis were performed using R version 4.1.0.<br>Two-step cluster analyses were conducted with SPSS 25 (SPSS, Chicago, IL, USA).

For manuscripts utilizing custom algorithms or software that are central to the research but not yet described in published literature, software must be made available to editors and reviewers. We strongly encourage code deposition in a community repository (e.g. GitHub). See the Nature Portfolio guidelines for submitting code & software for further information.

Data

Policy information about availability of data

All manuscripts must include a data availability statement. This statement should provide the following information, where applicable:

- Accession codes, unique identifiers, or web links for publicly available datasets
- A description of any restrictions on data availability
- For clinical datasets or third party data, please ensure that the statement adheres to our policy

Data from

Human research participants

Policy information about studies involving human research participants and Sex and Gender in Research.

Reporting on sex and gender	As this study didn't include human research participants, the sex and gender information of human research participants was not collected and considered.
Population characteristics	As this study didn't include human research participants, the information about the population characteristics of human research participants was not collected and considered.
Recruitment	As this study didn't include human research participants, the recruitment of human research participants was not conducted in the whole process of this study.
Ethics oversight	As this study didn't include human research participants, the organization approving the study protocol involving human research participants was not included.

Note that full information on the approval of the study protocol must also be provided in the manuscript.

Field-specific reporting

Please select the one below that is the best fit for your research. If you are not sure, read the appropriate sections before making your selection.

☐ Life sciences ☐ Behavioural & social sciences ☒ Ecological, evolutionary & environmental sciences

For a reference copy of the document with all sections, see nature.com/documents/nr-reporting-summary-flat.pdf

Ecological, evolutionary & environmental sciences study design

All studies must disclose on these points even when the disclosure is negative.

Study description	A synonymous mutation of Ubiquitin-specific Peptidase 15 (MvUSP15) was significantly associated with the physiological upper thermal limit of the mussel. The individuals carrying GG genotype (the G-type) at the mutant locus owned significantly lower heat tolerance compared to the individuals carrying GA and AA genotype (the A-type). Furthermore, when heated to sublethal temperature, the G-type exhibited higher inter-individual variations in the MvUSP15 expression, especially for the mussels on the sun-exposed microhabitats. Taken together, a synonymous mutation in MvUSP15 can affect the gene expression profile and interact with microhabitat heterogeneity to influence thermal resistance.
Research sample	The black mussel Mytilisepta virgata (Wiegmann, 1837), a native species widely-distributed in the Indo-west-Pacific, is supposed to be a model species for investigating fine-scale adaptive traits.<br>We collected the mussels in Dongshan Swire Marine Station (D-SMART), China (23.65°N, 117.49°E), in which two types of microhabitats coexisted at rocky shores.
Sampling strategy	A total of 187 mussels were randomly collected in the sun-exposed and shaded habitats along a 500 m shoreline in D-SMART on July 31st, 2021.
Data collection	The operative temperatures of M. virgata under filed conditions in the Dongshan Swire Marine Station (D-SMART), China (23.65°N, 117.49°E) were continuously recorded from July to August 2020 using biomimetic thermal loggers.<br>ddRADseq data was available (BioProject ID: PRJNA517974; BioSample accessions: SAMN10849586 - SAMN10849649).<br>The genotyping information of the 179 mussels was validated using Sanger sequencing.<br>The mRNA Expression of MvUSP15 was measured by quantitative real-time reverse transcription PCR.
Timing and spatial scale	A total of 187 mussels were collected in the sun-exposed and shaded microhabitats along a 500 m shoreline on July 31st, 2021.
Data exclusions	No data was excluded.
Reproducibility	The methods used are easily reproducible.
Randomization	We allocated mussels into heat-treated and control group randomly.
Blinding	We had no acquisition about the genotype of mussels when sampling.

Did the study involve field work? ☒ Yes ☐ No

Field work, collection and transport

Field conditions	The operative temperature of M. virgata was continuously recorded using biomimetic thermal loggers.
Location	Dongshan Swire Marine Station (D-SMART), China (23.65°N, 117.49°E), in which two types of microhabitats coexisted at rocky shores.
Access & import/export	Mytilisepta virgata is not protected animal, and the sampling site is not in reserve. Our sampling procedure was approved by D-SMART.
Disturbance	Only 187 mussels were collected from their habitats.

Reporting for specific materials, systems and methods

We require information from authors about some types of materials, experimental systems and methods used in many studies. Here, indicate whether each material, system or method listed is relevant to your study. If you are not sure if a list item applies to your research, read the appropriate section before selecting a response.

Materials & experimental systems

n/a	Involved in the study
☒	☐ Antibodies
☒	☐ Eukaryotic cell lines
☒	☐ Palaeontology and archaeology
☐	☒ Animals and other organisms
☒	☐ Clinical data
☒	☐ Dual use research of concern

Methods

n/a	Involved in the study
☒	☐ ChIP-seq
☒	☐ Flow cytometry
☒	☐ MRI-based neuroimaging

Animals and other research organisms

Policy information about studies involving animals; ARRIVE guidelines recommended for reporting animal research, and Sex and Gender in Research

Laboratory animals	The study did not involve laboratory animals.
Wild animals	Mytilisepta virgata; we caught the animals by knife; bring it back to the lab for acclimation.
Reporting on sex	The sex information of Mytilisepta virgata was not considered in this study, for the reason that we focus the thermal tolerance of mussels and there was no direct correlation between the thermal tolerance of mussels and their sex.
Field-collected samples	After collection, mussels were transported back to the laboratory within 3 h, placed in a plastic basket, and immersed in 20 l fresh seawater at a temperature of 22°C. During laboratory acclimation, mussels were fed concentrated microalgae every day over 2 months at water temperature of 21–22°C and salinity of 33 psu to simulate the in situ annual average water temperature. Seawater was aerated continuously and exchanged daily.<br>After acclimation, we killed the animals by putting them into liquid nitrogen container because we need adductor muscle to measure the expression levels of MvUSP15 gene.
Ethics oversight	Mytilisepta virgata is not protected animal, and the sampling site is not in reserve. Our sampling procedure was approved by D-SMART.

Note that full information on the approval of the study protocol must also be provided in the manuscript.
